# Clinical signs and symptoms of adult patients with intracranial arachnoid cysts

**DOI:** 10.1186/2045-8118-12-S1-O37

**Published:** 2015-09-18

**Authors:** Katrin KM Rabiei, Roberto Doria Medina, Mats Högfeldt, Per Hellström, Carsten Wikkelsö, Magnus Tisell

**Affiliations:** 1 Hydrocephalus research unit, Institution of Neuroscience, Sahlgrenska Academy, Sweden

## Background

Patients with arachnoid cysts have a wide range of symptoms from asymptomatic to a variety of symptoms and signs. Indication for surgical treatment can be challenging in case of the most common symptoms; headache and vertigo which are both subjective and common. The aim of this prospective study was to describe variety of symptoms in relation to cyst location and volume and their response to treatment.

## Methods

112 adults 46,6 y (18-77 y) with de novo cysts were prospectively included and evaluated through our hydrocephalus research unit with extensive test battery including neurological-, neuropsychological-, motor function testing and MRI. After evaluation, 76 patients were offered surgery. 54 accepted and 22 declined. 33 patients were regarded asymptomatic. Operated patients were followed up 3 months after surgery with the same test battery. Cyst volume was measured with OsiriX® software.

## Results

There was no significant difference in MMSE, frequency of head trauma or length of education between operated patients, those who declined or were asymptomatic. Asymptomatic patients had a significantly lower cyst volume, 18 ml (1-88), compared to those who were offered surgery 47 ml (3-223). Headache and dizziness was as prevalent in asymptomatic patients as the operated ones. In operated patients, headache improved significantly (p<0,05) 3 months after surgery. Cyst volume reduced to 31 ml (0-282, p<0,001) 3 months after surgery. Cyst volume reduced further to 29 ml (0-279, p<0,001) 1 year postoperatively. 43 patients considered themselves improved in at least one major symptom after 3 months. 9 patients were the same and 2 worse. In 22 (41%) patients at least one major symptom ceased completely. Clinical and radiological improvement did not correlate.

## Conclusion

80% of patients experienced an improvement in at least one major symptom 3 months after surgery. Clinical improvement did not correlate with reduction in cyst volume.

